# Defining the Infant Male Urobiome and Moving Towards Mechanisms in Urobiome Research

**DOI:** 10.21203/rs.3.rs-2618137/v1

**Published:** 2023-03-07

**Authors:** Maria Hadjifrangiskou, Seth Reasoner, Viktor Flores, Gerald Van Horn, Grace Morales, Leslie Peard, Benjamin Abelson, Carmila Manuel, Jessica Lee, Bailey Baker, Timothy Williams, Jonathan Schmitz, Douglass Clayton

**Affiliations:** Vanderbilt University Medical Center; Vanderbilt University Medical Center; Vanderbilt University Medical Center; Vanderbilt University Medical Center; Vanderbilt University Medical Center; Vanderbilt University Medical Center; Vanderbilt University Medical Center; Vanderbilt University Medical Center; Vanderbilt University Medical Center; Vanderbilt University Medical Center; Vanderbilt University Medical Center; Vanderbilt University Medical Center; Vanderbilt University Medical Center

**Keywords:** Urobiome, pangenome, urinary tract infection, pediatric urology, urinary microbiome

## Abstract

The urinary bladder harbors a community of microbes termed the urobiome, which remains understudied. In this study, we present the urobiome of healthy infant males from samples collected by transurethral catheterization. Using a combination of extended culture and amplicon sequencing, we identify several common bacterial genera that can be further investigated for their effects on urinary health across the lifespan. Many genera were shared between all samples suggesting a consistent urobiome composition among this cohort. We note that, for this cohort, early life exposures including mode of birth (vaginal vs. Caesarean section), or prior antibiotic exposure did not influence urobiome composition. In addition, we report the isolation of culturable bacteria from the bladders of these infant males, including *Actinotignum schaalii*, a bacterial species that has been associated with urinary tract infection in older male adults. Herein, we isolate and sequence 9 distinct strains of *A. schaalii* enhancing the genomic knowledge surrounding this species and opening avenues for delineating the microbiology of this urobiome constituent. Furthermore, we present a framework for using the combination of culture-dependent and sequencing methodologies for uncovering mechanisms in the urobiome.

## 1. Introduction

Until the past decade, it was presumed that the healthy urinary bladder was a sterile environment. However, advances in genetic sequencing and extended culture methodologies have uncovered a resident microbiota of the bladder, and this community has been termed the urobiome^1,2^. Since the discovery of the urobiome, several studies have demonstrated connections between urobiome dysbiosis and a variety of genitourinary diseases, including nephrolithiasis, recurrent UTIs (rUTIs), female urinary urge incontinence (UUI), male lower urinary tract symptoms (LUTS), and bladder cancer^3–7^. However, despite a decade of research on the urobiome, little progress has been made to understand the development of the urobiome and the mechanistic interactions of the urobiome with urinary pathogens.

To begin to address the question of urobiome development, several studies have investigated the urobiome of children^8–12^. However, these studies sampled pediatric subjects with a variety of pre-existing urinary tract diseases or infection, which was the indication for urinary catheterization. For a variety of other anatomic niches, including the skin and gastrointestinal tract, early life development of the resident microbiota shapes future microbial diversity and susceptibility to disease^13^. Therefore, defining the development of the healthy pediatric urobiome is a vital endeavor. To bridge this gap in the field and investigate the urinary microbiome of healthy infants, we collected catheterized urine samples under sterile operating room conditions at the time of circumcision of male infants under one year of age. Notably, none of the subjects had structural or functional urinary tract abnormalities, or prior urinary tract infection. Thus, our study represents the first investigation of the healthy infant urobiome, albeit limited to the male gender. We provide evidence of a detectable and culturable urobiome of healthy infant males. Using complementary approaches of extended culture and 16S rRNA amplicon sequencing, we report a diverse and consistent urobiome signature in infant males that does not appear to be perturbed by early life exposures, such as mode of delivery (vaginal vs. Caesarean section) or prior antibiotic exposure for non-urinary infections. Among the urobiome residents cultured, we report *Actinotignum schaalii* as a species of interest, because of its prevalence in both the adult and pediatric urobiomes^2,8,14–18^ and its implication as a uropathogen in certain patient populations^19–21^. To facilitate future mechanistic work on this urobiome member, we provide whole genome sequencing information of nine independent strains of *A. schaalii*.

Research investigations of the urobiome are still within their first decade. As ongoing research continues to define the urinary microbiome, standardized methods and reporting are vital^22^. Given the low biomass of the urinary microbiome, the potential for contamination during sample collection, processing and analysis is high. In this work, we utilize both culture-dependent and independent methodologies to assess the infant urobiome. We present rigorous sampling and processing controls to benchmark the potential contaminants introduced during sample collection and processing. We include extensive methodological, bioinformatic, and statistical documentation to promote accessibility and reproducibility within the nascent urobiome field.

## 2. Materials And Methods

### 2.1 Recruitment and Sample Collection

This study was approved by the Vanderbilt University Medical Center Institutional Review Board (IRB # 191815). Parental guardians provided written informed consent for sterile urinary catheterization under anesthesia in the operating room prior to the circumcision procedure. Exclusion criteria included structural or functional genitourinary abnormalities, prior urinary tract infection, or prior urethral catheterization. Urine samples were collected by transurethral catheterization following sterilization of the glans penis and foreskin. Urine samples were stored in sterile Falcon tubes and immediately placed on ice for transport to permanent storage. Within 2 hours of collection, urine samples were transferred to an −80°C freezer for indefinite storage.

### 2.2 Extended Culture

Prior to freezing urine samples, 100mL of urine was spread onto Columbia Agar with 5% Sheep Blood (BD BBL^™^ 221263) and Brucella Agar (Thermo Scientific^™^ R01255). Duplicate plates were incubated in aerobic and anaerobic conditions (anaerobiosis was attained using BD GasPak^™^ EZ anaerobe pouch system). Negative control plates of each respective agar were incubated simultaneously. Plates were incubated for up to 5 days at 37°C. Aerobic conditions included 5% CO_2_ atmosphere. Colonies were analyzed by MALDI-TOF (Bruker Daltonics), and colony identification performed pyrochemically by MALDI Biotyper^®^ (Bruker Corporation) with an extended research-use taxonomic library. Glycerol stocks were frozen for each unique colony isolated.

### 2.3 Amplicon 16S rRNA Sequencing

Urine samples were shipped on ample dry ice to University of California at San Diego Microbiome Center for DNA extraction and sequencing. DNA was extracted using the ThermoFisher MagMAX^™^ Microbiome Ultra Nucleic Acid Isolation Kit (A42357) from 500mL of sample. To benchmark DNA extraction efficiency, ten-fold serial dilutions of ZymoBIOMICS^™^ Microbial Community Standard (D6300) were extracted in parallel with urine samples. Following DNA extraction, the V4 hypervariable 16S rRNA region was amplified using the 515F and 806R primers from the Earth Microbiome Project^23^. To benchmark PCR amplification of 16S rRNA, ten-fold serial dilutions of ZymoBIOMICS^™^ Microbial Community DNA Standard (D6306) were amplified in parallel with extraction standards and urine samples. Additionally, negative control wells (extraction blanks) lacking sample input were subjected DNA extraction, PCR amplification, and sequencing. Further description of the methods and controls is available in the Supplementary Methods.

### 2.4 16S rRNA Sequencing Analysis

All sequencing processing and analyses were completed in R (version 4.2.1). Sequences were processed using DADA2 to trim, filter, learn error rates, denoise, merge, and remove chimeras from reads^24^. Amplicon sequence variants (ASVs) were assigned to merged reads with the SILVA rRNA database (version 138.1) using the DADA2 function assignTaxonomy. ASVs were merged with their taxonomy in the R package phyloseq^25^. The R package Decontam was used to identify and remove potential contaminant ASVs using the prevalence method and a threshold of 0.3. This threshold was chosen after evaluating the removal of contaminating ASVs of the ZymoBIOMICS^™^ Microbial Community Standard dilution series (Supplementary Methods). The R packages microbiome, microViz, and vegan were used to format and visualize the 16S rRNA data.

### 2.5 DNA Extraction from *Actinotignum schaalii* and Whole Genome Sequencing

Colonies of *A. schaalii* were resuspended in PBS, lysed with a combination of lysostaphin, lysozyme, and Proteinase K at 37°C. Next, the suspension was treated with RNase. Following dilution of the lysate in H_2_O, the mixture was sonicated at 34 kHz for 4 minutes. DNA was purified with three successive extractions in phenol:chloroform:isoamyl alcohol (25:24:1). Finally, DNA was precipitated from ethanol and resuspended in H_2_O. DNA was sent on dry ice to SeqCenter (formerly, Microbial Genomic Sequencing Center, Pittsburgh, PA). Sample libraries were prepared using the Illumina DNA Prep kit and IDT 10bp UDI indices, and sequenced on an Illumina NextSeq 2000, producing 2×151bp reads. Demultiplexing, quality control and adapter trimming was performed with bcl-convert (v3.9.3).

### 2.6 *Actinotignum schaalii* Sequencing Analysis

Trimmed reads were assembled into contigs >1000bp using Shovill (v. 1.1)^26^. Contigs were annotated with Bakta using default settings^27^. Annotated files were analyzed by Roary to construct the core genome and pangenome^28^. Anvi’o was used to visualize the pangenome, calculate average nucleotide identity, and visualize a phylogenetic tree^29,30^. Contigs were reformatted into anvi’o format and annotated with the COG20 database^31^. Functional enrichment of the core and accessory genomes were calculated using anvi’o pangenome summarize function. ABRicate was used to determine the presence of antimicrobial resistance genes and virulence factors on the unannotated contigs. ResFinder (v. 4.0), MegaRes (v. 3.0), and VirulenceFinder Database (VFDB, v. 5). were used as the reference databases of ABRicate. Contigs were uploaded to the antiSMASH online interface and analyzed with antiSMASH beta version 7.0 which includes an updated algorithm for the prediction of non-ribosomal peptide produced metallophores^32,33^. antiSMASH settings were relaxed detection strictness, and KnownClusterBLAST, MIBig cluster comparison^34^, and Cluster Pfam analysis.

## 3. Results

**Figure 1:**
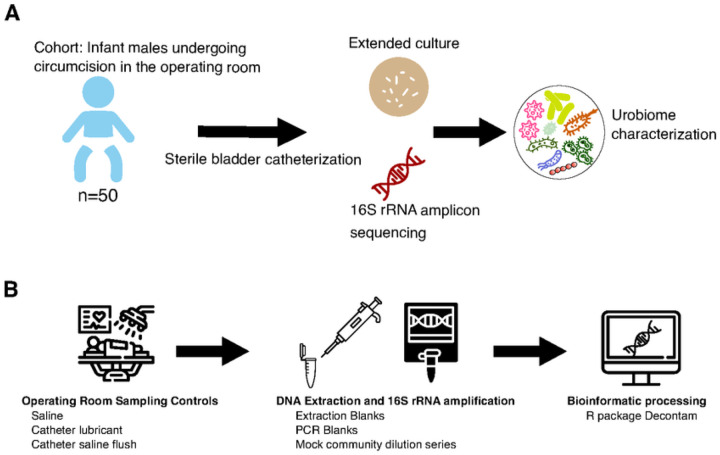
Study Schematic and Analysis Workflow. **A)** Illustration of study design. Fifty male infants were sterilely catheterized in the operating theatre prior to undergoing circumcision. Urine was immediately plated for extended quantitative urine culture (EQUC). DNA was extracted from urine samples, amplified with V4 16S rRNA primers, and sequenced using Illumina paired-end chemistry. The combination of urine culture and sequencing results was used to describe the urobiome composition. **B)** Illustration of analysis workflow and evaluation of potential contaminant sources. Sampling controls were collected contemporaneously with urine samples in the operating theatre. Extraction blanks and a mock microbial community dilution series were used to benchmark DNA extraction. No template blanks were subjected to 16S rRNA PCR amplification to benchmark PCR amplification. All controls mentioned were subjected to Illumina paired-end sequencing. The Decontam package in R was used to filter potential contaminant sequences.

This study aimed to bridge a gap in our knowledge of the healthy pediatric urobiome. Our study prospectively enrolled 50 healthy male infants that underwent urinary catheterization during routine operative circumcision (Figure 1A). Below, we describe the pipeline we established to increase rigor and reproducibility within urobiome research, followed by a description of our findings.

### Establishing Methodology for Low Biomass Urine Samples from Infants

The method of sample collection is a key concern in urobiome research. Genital and intestinal contaminants confound urobiome results^22^. To obtain sterile catheterized samples from healthy infants, we selected the population of infant males undergoing circumcision in the operating room. Informed consent for bladder catheterization was obtained from parental guardians. We collected urine from 50 male infants following induction of general anesthesia and sterilization of the periurethral area. The median age of the infants was 215 days (~7 months old, Table 1). The average amount of urine collected was 5.81 mL (range 0.4–28mL). Urine was immediately plated for extended quantitative urine culture (EQUC) as described in the methods to isolate and identify culturable bacteria. For sequencing, aliquots of the same urine were immediately frozen at −80°C to prevent microbial growth or contamination prior to processing for sequencing.

We modified existing EQUC urobiome protocols to preserve urine volume for DNA extraction^2^. We utilized two non-selective agar media (blood agar and Brucella agar) plated in duplicate and incubated under aerobic and anaerobic conditions. Aliquots of the same urine were subjected to DNA extraction using a commercially available kit which utilizes bead beating and DNA binding by magnetic beads for DNA isolation and purification. Isolated DNA was amplified using standardized PCR primers for the V4 region of the 16S rRNA. 16S rRNA amplicons were sequenced by Illumina paired-end sequencing.

The urobiome is a low biomass environment. There are myriad potential sources of contamination, a concern which is accentuated for low biomass samples. Contamination can be introduced at any step of sample processing, from sample collection to DNA extraction and amplification to sequencing^35–37^. We utilized three types of negative controls: 1) DNA extraction blanks; 2) no template PCR amplification blanks; and 3) sampling controls (Figure 1B). Specifically, we included eight DNA extraction blanks which underwent all steps of DNA extraction, PCR amplification, and sequencing. We included four no DNA template blanks during PCR amplification of the V4 region of the 16S rRNA. Finally, we included three types of sampling controls (operating theatre saline, mineral oil used for catheter lubrication, and saline flushed through a sterile catheter); four sets of which were collected on separate days. To our knowledge, this is the first urobiome study to report sampling controls collected contemporaneously with urine samples.

**Figure 2:**
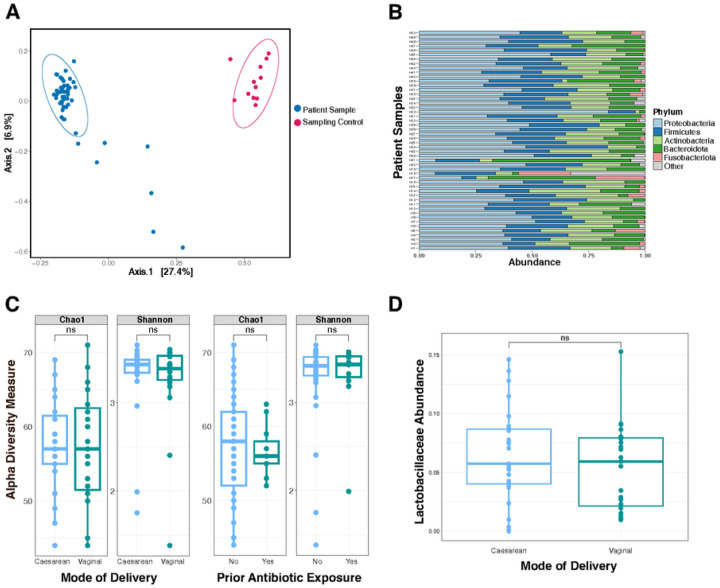
16S rRNA Amplicon Sequencing Reveals a Consistent Urobiome Composition. **A)** Beta diversity between infant urine samples and sampling controls. Beta diversity was calculated by the phyloseq “ordinate” function using Bray-Curtis distances. Urine samples were significantly different than sampling controls by permutational multivariate analysis of variance (PERMANOVA, p=0.001). PERMANOVA was calculated using the vegan function “adonis2”. **B)** Phyla-level taxonomic profiles of urine samples from 50 infants. Urine samples are depicted along the vertical axis and taxonomic relative abundance on the x-axis. Plot created with the microViz function “comp_barplot”. **C)** Alpha diversity metrics (Shannon index and Chao1) between urine from infants born by vaginal delivery vs. Caesarean section (left); and between urine from infants previously exposed to antibiotics vs. antibiotic naïve (right). Alpha diversity was calculated within the phyloseq package using the “plot_richness” function. Alpha diversity was not significant different between groups by Wilcoxin rank sum test (p>0.05). **D)** Relative abundance of Lactobacillaceae in urine samples between infants born by vaginal delivery vs. Caesarean section. The Lactobacillaceae family was agglomerated with the phyloseq command “tax_glom”. There was no significant difference in Lactobacillaceae abundance between groups by Wilcoxin rank sum test (p>0.05).

We sequenced sampling controls on an Illumina NovaSeq 6000 to add additional resolution to rare contaminant sequences and due to sequencing equipment availability. Urine samples were sequenced on an Illumina MiSeq. Notably, all samples and controls were processed in the same laboratory using the same reagents. To account for the different read numbers between MiSeq and NovaSeq platforms, we utilized a dilution series of a mock microbial community. Sampling controls had consistently higher 16S rRNA reads than extraction blanks. All sample read counts are shown in Supplementary Table S1. We applied the R package Decontam to remove sequences that were more prevalent in the blank extraction controls or PCR blanks compared to the sampling controls. A total of 37 specific genera were retained following filtering using Decontam. The most prevalent genera present in the sampling controls were *Campylobacter, Rodentibacter, Mannheimia, Alloprevotella* (Supplementary Table S2). The sampling controls (n=12) clustered distinctly from the subjects’ samples (n=50) (PERMANOVA p=0.001) (Figure 2A). Nonetheless, the number of 16S rRNA reads in sampling controls indicates that this is a potential source of contamination that must be accounted for within urobiome studies.

### Characterizing the Urobiome by Amplicon Sequencing

Prior urobiome studies^2,38^ have used agarose gel electrophoresis to determine “negative samples” following 16S rRNA amplification (and thus excluded those samples from sequencing). The absence of a band in gel electrophoresis to determine negative samples has a false negative rate of 30% and should be avoided when sampling low biomass environments^39^. We subjected all samples to 16S rRNA amplification and sequencing. Every subject’s sample had higher sequencing reads than blank extraction controls. The range of merged non-chimeric 16S rRNA reads in the subjects’ samples from Illumina MiSeq sequencing was 6604–60040 reads compared to 2345–4149 reads in the extraction blank controls (Supplementary Table S1). We used the R package Decontam to identify potential contaminants which were sequences more prevalent in the negative controls than the urine samples. Given the different library sizes and readily apparent compositional differences (Figure 2A), we did not apply Decontam to remove sampling controls from subject samples. Next, we filtered ASVs less than 1% abundance in the whole subject dataset. This threshold has previously been applied to urobiome datasets^7^. Supplementary Table S3 includes all taxa from subject urine samples identified by 16S rRNA sequencing with literature citations regarding prior detection in urobiome studies.

Following above-described filtering steps, there were 74 unique taxa remaining from the urine samples (Supplementary Table S4). Consistent with prior reports, the phyla Proteobacteria, Firmicutes, Bacteroides, and Actinobacteria were frequently detected (Figure 2B)^40^. The genera shared between subject samples and sampling controls were *Campylobacter, Staphylococcus, Nocardiopsis, Halomonas, Saccharopolyspora, Vibrio, Porphyromonas, Rheinheimera, Cloacibacterium*, and *Anaerobacillus*. Urine samples had a median of 41 unique genera (range 32–57). Six genera were detected in all 50 subject urine samples: *Staphylococcus, Nocardiopsis, Acinetobacter, Pseudomonas, Corynebacterium*, and *Nesterenkonia*. Three genera were found in 49 of the 50 samples: *Aliihoeflea, Saccharopolyspora*, and *Sphingobacterium*. Three genera were found in 48 of the 50 samples: *Escherichia-Shigella, Lactobacillus*, and *Halomonas*. The most abundant genera were *Nocardiopsis, Staphylococcus, Escherichia-Shigella* (median abundance >5%); five additional genera had median abundance >3%: *Lactobacillus, Acinetobacter, Pseudomonas, Prevotella*, and *Lacibacter*.

We sought to investigate whether various subject exposures influenced the diversity of the urobiome. Measures of community diversity are commonly used to summarize information about the richness and distribution of microbials species in the community^41^. We compared two measures of alpha diversity (Chao1, Shannon) for two subject exposures: mode of birth (vaginal delivery vs. Caesarean section) and prior antibiotic exposure (Figure 2C). While both of these exposures are known to alter the gastrointestinal and skin microbiota of infants^42^, no significant difference in alpha diversity was detected in the urine samples between either exposure (Figure 2C).

Next, we sought to determine whether specific taxa are influenced by subjects’ exposures. We selected the taxonomic family Lactobacillaceae which was present in variable amounts in the 50 subjects.Lactobacillaceae are well studied members of the urogenital microbiota, particularly in post-pubescent women^43,44^. Lactobacillaceae are transferred to infants during vaginal birth, and intestinal abundance of Lactobacillaceae are decreased in infants born by Caesarean section^45^. We compared Lactobacillaceae abundance between infants born by vaginal birth *vs*. Caesarean section (Figure 2D). There was no significant difference inLactobacillaceae abundance between these groups. Together, these data display a detectable and consistent urobiome among infant males. Twelve genera were detected in ≥48 of the 50 urine samples. Early life exposures, such as mode of birth and prior antibiotic exposure, did not significantly influence urobiome composition.

### Expanded Quantitative Urine Culture Identifies Culturable Members of the Infant Urobiome

To facilitate future mechanistic studies between urobiome members and the urothelium, or uropathogenic bacteria, we designed an extended quantitative urine culture (EQUC) protocol with the goal of capturing as many bacteria as possible using limited urine volume from infants. Indeed, 32/50 (64%) of urine samples led to identifiable growth on one or more of the media and conditions. This percentage is consistent with several prior urobiome studies utilizing extended culture across the human lifespan^1,2,8,14^. Colony identification was performed by matrix-assisted laser desorption/ionization (MALDI) mass spectrometry. Among the 12 sampling controls, only 1 colony grew from extended culture, *Cutibacterium acnes*, a likely skin contaminant. This suggests that the 16S rRNA reads observed in the sampling controls were due to residual DNA, not viable bacteria.

The species identified by extended culture are listed in Table 2. The range of unique species was 1–5 per urine sample. The most common taxonomic families detected were Actinomycetaceae (n=15), Peptoniphilaceae(n=7), and Enterococcaceae(n=6). The most common species isolated were *Actinotignum schaalii* (n=9), *Enterococcus faecalis* (n=6), and *Peptoniphilus harei* (n=5).

**Figure 3:**
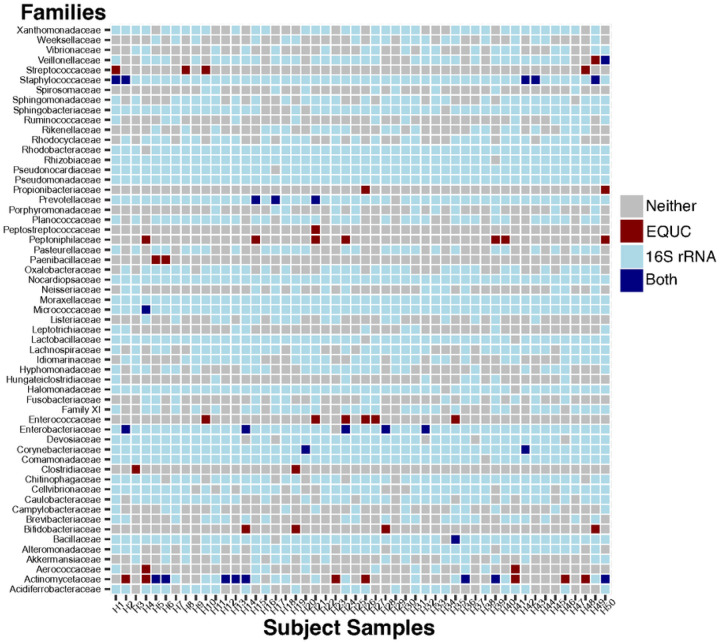
Concordance between EQUC and Amplicon Sequencing Results. Co-occurrence detection patterns of taxonomic families between EQUC and amplicon sequencing methodologies. Taxonomic families are arranged vertically and patient samples horizontally. The rectangles indicate the detection of the family by EQUC (maroon), 16S rRNA amplicon sequencing (light blue), both methodologies (dark blue), or neither methodology (gray).

Extended culture and amplicon sequencing are complementary but not strictly equivalent approaches. We created a concordance map that displays which taxonomic families were detected by EQUC, 16S rRNA sequencing, or both (Figure 3). We included families detected in >0.1% relative abundance in the 16S rRNA dataset. There were a total of 49 taxonomic families across the 50 subjects’ urine samples. The family Actinomycetaceae was the most frequently detected family by EQUC and exhibited a high level of concordance with 16S rRNA results.

We inspected the concordance map for taxonomic families disproportionately represented in either EQUC or 16S rRNA results. The families Moraxellaceae, Nocardiopsaceae, Pseudomonadaceae were detected in all urine samples by 16S rRNA amplicon sequencing, but not by EQUC. Similarly, the physiologically important family Lactobacillaceae was frequently detected by amplicon sequencing but not isolated by EQUC. The families Bifidobacteriaceae, Enterococcaceae, Peptoniphilaceae, Streptococcaceae were detected >3 times by EQUC but not present in the 16S rRNA results. These discordances highlight potential limitations of each method and the importance complementary approaches for sampling the urobiome.

### *Actinotignum schaalii* is a Common Culturable Constituent of the Infant Urobiome

*Actinotignum schaalii* was the most common species identified in our extended culture and exhibited high concordance with the 16S rRNA amplicon sequencing results. Of the 32 urine samples that grew at least one bacterial species, nine (28.1%) grew *A. schaalii. A. schaalii* (formerly *Actinobaculum schaalii*) has been detected in numerous urobiome studies to date^2,8,14–18,46^. Intriguingly, in addition to being reported in this study and others as an asymptomatic colonizer of the urobiome, *A. schaalii* is also an opportunistic causative agent of urinary tract infections^47^. Specifically, there is concern of an increasing incidence of *A. schaalii* urinary tract infections^19–21^. Given the relatively fastidious growth requirements of *A. schaalii*, standard clinical microbiological techniques may not detect *A. schaalii* from urine samples^21,48,49^, highlighting the need to broaden our understanding of *A. schaalii* in the urinary tract. To date, genome analysis of *A. schaalii* has been limited to genome announcements without comprehensive analysis^50^. To expand our understanding of *A. schaalii*, we performed whole-genome sequencing on nine separate *A. schaalii* isolates identified by extended culture of urine from male infants.

**Figure 4:**
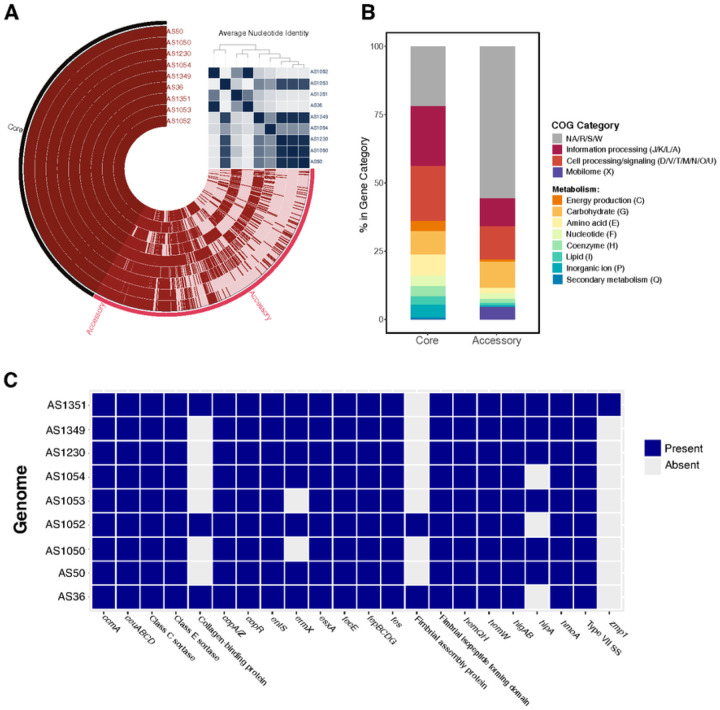
Genomic Characterization of *Actinotignum schaalii* Isolates. **A)** Nine *A. schaalii* genomes isolated by EQUC from distinct subjects were subjected to whole genome sequencing. *A. schaalii* pangenome of the nine isolates visualized using anvi’o. Core genes were present in 100% of isolates (9/9) while the accessory genome consists of gene present in <9 of the genomes. Clustering of the genomes is based on average nucleotide identity (ANI), shown in the upper right matrix. **B)** Relative abundance of COG categories represented in the core and accessory genomes. **C)** Presence-absence matrix of fitness factors and antimicrobial resistance genes. ABRicate was used to screen contigs using the MegaRes, ResFinder, and Virulence Factor databases.

We generated high quality whole genome sequences of each *A. schaalii* isolate, with a mean Q30 sequencing coverage of 275x. The mean genome length was 2,325,278 bp with an average of 1931 coding sequences (CDS). Following annotation of genes with Bakta^27^, we computed the pangenome with Roary^28^. The core genome shared by all nine isolates was composed of 831 genes. An additional 2081 genes were found in 2–8 of the isolates. Finally, there were 1626 unique genes found in only 1 of the nine isolates. We visualized the pangenome and calculated average nucleotide identify (ANI) with anvi’o (Figure 4A). We compared the gene clusters in the core and accessory genomes using by annotating clusters by COG category within anvi’o. Overall, 21.9% of the core genome and 55.8% of the accessory genome were classified as general functions (R), unknown functions (S) or unassigned within the COG database (NA) (Figure 4B). The core genome was enriched for genes involved in information processing (DNA replication, transcription, etc.; COG J/K/L/A), cell processing/signaling (COG D/V/T/M/N/O/U), and energy production (COG C). Interestingly the accessory genome was enriched for genes involved in carbohydrate metabolism (COG G) Intuitively, gene clusters involved in mobile gene transfer (COG X) were elevated in the accessory genome (4.6% vs. 0.06%), consistent with the flexible nature of the accessory genome.

To identify potential determinants of *A. schaalii* fitness in the urinary tract, we used ABRicate to screen for the presence of antimicrobial resistance genes and known fitness factors, utilizing the ResFinder, MegaRes, and the VirulenceFinder Database. Notably, the Actinomycetaceae family is poorly represented in the VFDB and genomic datasets in general^51^, limiting the identification of putative virulence factors. We also annotated contigs with Bakta which reduces the number of CDS annotated as hypothetical proteins^27^. We manually curated potential fitness factors from the Bakta annotations. Seven of the nine isolates encoded *ermX*, an rRNA methyltransferase conferring resistance to macrolides. All nine isolates encoded the Esx-1 Type VII secretion system and its toxin *esxA* (Figure 4C). Esx-1 has been most extensively characterized in *Mycobacterium tuberculosis*, a member of the phylum Actinobacteria like *A. schaalii*^52,53^. EsxA is an anti-eukaryotic membrane-permeabilizing toxin and is required for virulence in *M. tuberculosis*^53^.

Metal acquisition and homeostasis are key fitness determinants for microbial-host interactions^54,55^. All nine isolates contained the enterobactin transporters, *entS* and *fepBCDG* (Figure 4C). The siderophore enterobactin, an iron-chelating small molecule, is a known fitness factor within the iron-deplete urinary tract^56^. Analysis of *A. schaalii* contigs using antiSMASH^32,33^ to identify biosynthetic gene clusters (BGCs), particularly those responsible for siderophores production, did not reveal any putative BGCs that may produce enterobactin or related molecules (Supplementary Table 5). Systems for the acquisition and metabolism of heme were also ubiquitous in the 9 isolates. Specifically, all nine isolates encoded *hemQ* and *hemH* (coproheme decarboxylase and ferrochetalase, respectively) which are involved in heme biosynthesis, the heme chaperone *hemW*, the heme ATPase transporter *ccmA*, and the heme-degrading monooxygenase *hmoA*. Furthermore, all nine isolates encoded copper detoxification systems. Copper is toxic to bacteria in high concentrations and is elevated in the urinary tract during infection^57,58^. Thus, copper detoxification is considered a fitness factor in the urinary tract. All nine isolates encoded *copA/Z*, a copper exporter and chaperone respectively, and *copR*, a copper responsive transcriptional regulator (Figure 4C). Together, these results indicate that *A. schaalii* encodes known fitness factors within the phylum Actinobacteria (*e.g*. EsxA) and within disparately related urinary pathogens (metal acquisition and detoxification).

## 4. Discussion

The importance of understanding how the microbiome of a given anatomic niche shapes the biology and health of a host has never been more critical. In the genitourinary tract it is now well-accepted that a urobiome exists and plays a role in several urologic conditions^3–7^. Yet, compared to other anatomic niches, like the oral cavity, the gut and the skin, research surrounding the microbiome of the bladder is at its infancy. We use complementary approaches of extended culture and 16S rRNA amplicon sequencing to identify bacteria in catheterized urine samples from healthy infant males. With EQUC, we isolated 43 unique bacterial species and 64% of urine samples grew at least one colony by EQUC (Table 2), a percentage consistent with many prior urobiome studies^1,2,8,14^. These patient-derived isolates open exciting avenues for studying the interactions of urobiome members.

Our study reveals twelve genera (*Staphylococcus, Nocardiopsis, Acinetobacter, Pseudomonas, Corynebacterium, Nesterenkonia, Aliihoeflea, Saccharopolyspora, Sphingobacterium, Escherichia-Shigella, Lactobacillus*, and *Halomonas*) that were detected by 16S rRNA amplicon sequencing in ≥48 of the 50 urine samples. This indicates a consistent urobiome composition among healthy infant males. Urobiome alpha diversity was not significantly different between infants born by vaginal vs. Caesarean section, nor was diversity affected by prior antibiotic exposure (Figure 2C).

Males younger than one-year-old have higher rates of urinary tract infection (UTI) than females^59^. Various explanations for this difference have been proposed, including hormone levels and lack of circumcision^60,61^. The abundance of the taxon Enterobacteriaceae, the predominant cause of UTIs, in our dataset (Figure 3) may offer an additional exploratory hypothesis for the higher rates of UTIs in male infants. As previously noted, none of the subjects in this study had prior UTI. Notably, in our cohort, urobiome alpha diversity was not significantly different between infants born by vaginal vs. Caesarean section, nor was diversity affected by prior antibiotic exposure (Figure 2C). This could be due to several reasons: the median age of the male subjects was 7 months old; it is possible that any differences in urobiome composition arising from different modes of delivery have not persisted over time. Another possibility is that once the urobiome is established, it is not perturbed by diet, given the limited metabolites that are excreted in the urine compared to the gut. Likewise, depending on the antibiotic class, dosage and duration of course, antibiotic concentrations in the urine may not have been sufficient to leave a lasting imprint on the urobiome.

Interestingly, amplicon sequencing did not detect the genus *Porphyromonas* in high abundance, nor did any samples grow *Porphyromonas* on EQUC (Porphyromonadaceae in Figure 3). *Porphyromonas* has been previously described as a major component of the male pediatric urobiome from voided urine samples^12^. Our analysis and another pediatric urobiome study^62^ did not detect *Porphyromonas* in catheterized samples, suggesting that *Porphyromonas* may originate from the urethra and not the bladder. This observation is supported by urethra-specific sampling in adult males^63^.

The family Nocardiopsaceae, specifically the genus *Nocardiopsis*, was frequently identified in our 16S rRNA data (Figure 3). The closely related genera *Nocardioides* has been detected in several urobiome studies^38,64^. Still, soil and water bacteria, like *Nocardiopsis*, are well described contaminants of laboratory supplies and reagents. Our methods for filtering contaminants did not remove *Nocardiopsis* from our subjects’ samples. This requires further attention to determine whether *Nocardiopsis* may be a yet unculturable member of the urobiome or an unfiltered sequencing contaminant.

As ongoing research continues to define the urinary microbiome, standardized methods and reporting are vital^22^. Given the low biomass of the urinary microbiome, the potential for contamination is high. We utilized rigorous sampling and processing controls to benchmark the potential contaminants introduced during sample collection and processing. Importantly, this study sets the precedent of collecting and reporting urinary catheter controls as a potential source of contamination. Following sampling of more catheter types and collection environments, the contaminant identification package SourceTracker may become useful within the urobiome field^65^. The analysis of urobiome amplicon sequencing data must account for the low biomass of this sample type and potential sources of contamination. Thus far, reporting of analysis parameters and filtering thresholds has been insufficient for the replication of these studies. To promote reproducibility within the urobiome field, we have included extensive methodological and bioinformatic detail herein. We hope this resource will improve the reproducibility of amplicon sequencing analysis by the urobiome field.

Finally, thus far, urobiome studies have been predominately descriptive studies. Using *Actinotignum schaalii* as a representative example, we show how the complementary approaches of extended culture and sequencing can uncover exploratory hypotheses by which bacteria may colonize and opportunistically infect the urinary tract. Using whole genome sequencing of nine *A. schaalii* genomes isolated by extended culture, we identified that *A. schaalii* possesses the transporters for enterobactin uptake but not the biosynthetic machinery for its production. This intriguing observation raises the questions of whether *A. schaalii* produces yet unidentified siderophores or whether *A. schaalii* may utilize siderophores produced by other urobiome constituents, known as xenosiderophore scavenging. These observations produce a testable hypothesis for the interactions of *A. schaalii* with other members of the urobiome community.

In summary, our study provides a snapshot of the pediatric urobiome of healthy infant males. From extended culture, we create an inventory of cultured urobiome constituents for future mechanistic studies. Finally, we report a comprehensive map of genomic features for the urobiome resident *A. schaalii* that appears to exhibit both commensal and uropathogenic properties in humans.

## Figures and Tables

**Table 1.** Cohort Details

**Participants (*n*)**	50
**Median age (days) at Sample Collection (IQR)**	215 (190, 252)
**Male sex**	100%
	
**Birth History**	
Caesarean section	27 (54%)
Preterm	18 (36%)
NICU following birth	18 (36%)
	
**Health Exposures**	
Prior antibiotic exposure	12 (24%)
	
**Type of Nutrition**	
Breast Milk only	5 (10%)
Formula	19 (38%)
Breast Milk and Formula	15 (30%)
Solids or Puree	11 (22%)
**Urine Collection**	
Volume of urine (mean, range)	5.81 mL (0.4–28 mL)

**Table 2.** Extended Quantitative Urine Culture

Species	Number of Isolates
*Actinomyces europaeus*	1
*Actinomyces naeslundii*	1
*Actinomyces odontolyticus*	1
*Actinomyces radingae*	1
*Actinomyces turicensis*	2
*Actinotignum schaalii*	9
*Aerococcus urinae*	1
*Alloscardovia ommnicolens*	1
*Anaerococcus* spps.	1
*Bacillus cereus*	1
*Bifidobacterium breve*	1
*Bifidobacterium dentium*	1
*Bifidobacterium longum*	1
*Citrobacter koseri*	1
*Clostridium sordelli*	1
*Clostridium tertium*	1
*Corynebacterium aurimucosum* group	1
*Corynebacterium* spps.	1
*Cutibacterium acnes*	2
*Enterobacter aerogenes*	1
*Enterococcus faecalis*	6
*Escherichia coli*	2
*Finegoldia magna*	1
*Klebsiella oxytoca*	1
*Murdochiella asaccharolytica*	2
*Paenibacillus* spps.	1
*Peptoniphilus harei*	5
*Peptostreptococcus anaerobius*	1
*Prevotella corporis*	1
*Prevotella* spps.	1
*Prevotella timonensis*	1
*Proteus mirabilis*	1
*Rauotella ornithinolytica*	1
*Rothia aeria*	1
*Staphylococcus aureus*	1
*Staphylococcus capitis*	1
*Staphylococcus haemolyticus*	1
*Staphylococcus hominis*	2
*Streptococcus mitis oralis*	3
*Streptococcus salivarius*	1
*Streptococcus vestibularis*	1
*Trueperella bernardiae*	1
*Veillonella parvula* group	2

## Data Availability

All sequence data derived from this work are publicly available in NCBI-Genbank databases under Bioproject PRJNA912725. Accession numbers are listed within Supplementary Table S6. All code used for bioinformatic analysis is publicly available within the supplementary methods: https://github.com/reaset41/Infant-Urobiome.
